# Effect of Avatar Head Movements on Communication Behavior and Subjective Evaluations of Presence and Success in Triadic Conversations

**DOI:** 10.1177/23312165261432065

**Published:** 2026-04-08

**Authors:** Angelika Kothe, Volker Hohmann, Giso Grimm

**Affiliations:** 1 Department of Medical Physics and Acoustics, 597451 Carl von Ossietzky Universität Oldenburg, and Cluster of Excellence “Hearing4All”, Oldenburg, Germany

**Keywords:** communication behavior, conversation success, head movement, audiovisual virtual reality, hearing device evaluation

## Abstract

Interactive communication in virtual reality can be used in experimental paradigms to increase the ecological validity of hearing device evaluations. This requires the virtual environment to elicit natural communication behavior in listeners. This study evaluates the effect of virtual animated characters’ head movements on participants’ communication behavior and experience. Triadic conversations were conducted between a study participant and two confederates. To facilitate the manipulation of head movements, the conversation was conducted in telepresence using a system that transmitted audio, head movement data and video with low delay. The confederates were represented by virtual animated characters (avatars) with different levels of animation: Static heads, automated head movement animations based on speech level onsets, and animated head movements based on the transmitted head movements of the confederates. A condition was also included in which the live videos of the confederates’ heads were embedded in the visual scene. Sixteen young adults (19–32 years) with self-reported normal hearing participated in the study, i.e., 16 triads were measured. The results show significant effects of animation level on the participants’ speech and head movement behavior as recorded by physical sensors, as well as on the subjective experience. The largest effects were found for the range of head orientation while speaking, the head orientation while listening, and the perceived realism of avatars. We therefore conclude that the representation of conversation partners affects communication behavior, which may be considered when natural speech and movement behavior is desired.

## Introduction

Face-to-face conversations in noisy environments occur frequently in every-day life (Wolters et al., 2016). They are an important part of social interaction, and frequent misunderstandings negatively impact self-confidence, social participation and overall well-being in people across the lifespan (Hogan et al., 2015; Hoppe et al., 2014; Punch & Hyde, 2011). For hearing-impaired listeners, verbal conversations in groups are typically challenging, especially when background noise is present. Hearing devices aim to increase speech intelligibility and reduce listening effort, but about 25% of users continue to report low satisfaction with their device in difficult listening situations, according to large-scale surveys (Carr & Kihm, 2022; Kim et al., 2022). Especially for patients with higher degrees of hearing loss, current hearing devices provide insufficient support (Hoppe et al., 2014; Punch & Hyde, 2011).

Improving device performance is not simple, as it requires identifying and attenuating irrelevant signals in diverse and dynamic sound scenarios. This task may be facilitated by recent developments that use the user's gaze and head movement behavior to distinguish between relevant and irrelevant sources (Best et al., 2017; Favre-Félix et al., 2018; Grimm et al., 2018). Such behavior-based signal processing strategies require systematic tests that elicit natural behavior. For example, interactive conversation was found to elicit different head movement behavior from the listener than isolated listening (Hadley et al., 2019; Hartwig et al., 2021). It has been suggested that widely used evaluation methods and measures, such as speech reception thresholds, are poor predictors of device performance in real life when used to evaluate more complex algorithms (Keidser, 2016; Naylor, 2016). This may be partly due to the fact that the head movement behavior of listeners in these traditional methods does not reflect real life behavior. One way of overcoming the limitations of traditional evaluation methods is to base evaluations of behavior-controlled hearing devices on interactive conversations (Grimm et al., 2023). In real-world environments, undisturbed audio signals cannot be accessed directly, and experiments are difficult to reproduce due to varying environmental conditions. Conversation-based paradigms for evaluating hearing devices can be performed in more controlled conditions using virtual audio-visual environments in the laboratory, thereby increasing precision and reproducibility.

In face-to-face conversations, non-verbal cues play a significant role. For instance, speakers’ head and eyebrow movement has been demonstrated to enhance speech understanding by aligning with prosodic cues (Graf et al., 2002). Munhall et al. (2004) found that natural head movements facilitate syllable identification. In addition, head orientation may support turn-taking, for example by visually indicating the intended addressee of a question. Furthermore, head movement and eye contact were found to facilitate communication and establish a sense of comfort (Aburumman et al., 2022; Rogers et al., 2022).

Given the critical role of non-verbal cues in real conversations, their simulation in virtual environments becomes essential for maintaining ecological validity in experimental paradigms. When moving from face-to-face conversations to virtual conversation scenarios, the influence of non-verbal behavior can still be present even when the interlocutors are represented by animated avatars. Hendrikse et al. (2018) showed that the listener's head movement behavior depends on the level of lip movement and head orientation animation of virtual animated characters when following a conversation. It was also found that manipulating the head movements of an interlocutor in an active conversation changes the movements of the receiver (Boker et al., 2009). Therefore, to achieve high ecological validity in virtual experiments, it is crucial to accurately represent interlocutors’ non-verbal cues—such as head orientation, gaze, and nodding—within the virtual environment.

Our overarching aim is to provide paradigms for the evaluation of hearing devices during interactive verbal conversation, based on physical measures of communication behavior, such as head movement, gaze or speech features. This work specifically aims to contribute to current research by investigating the effects of head movements of avatars on the listener's behavior and experience in order to assess their relevance in interactive scenarios. The research question of this study is:

### Does the Realism of Head Movements of Avatars Affect Behavior and Experienced Involvement in Virtual Environment Scenes?

Our approach involved observing participants in real triadic conversations. Triadic conversations are the minimum configuration in which turn-taking results in horizontal head movement. They are also a common scenario in everyday life (Peperkoorn et al., 2021). To systematically assess the effect of head movements on conversational behavior, the conversation was conducted via telepresence between a study participant and two confederates. The confederates were represented by avatars with varying levels of animation. These avatars were displayed on a projection screen using virtual animated characters. Two levels of background noise were used to control the level of difficulty. The measurement environment was designed to resemble a typical pub conversation, both visually and acoustically. To investigate the effects on a reference group, the participants were young people with reported normal hearing.

We expected that animating head movements in a way to provide realistic non-verbal cues would facilitate the conversation by supporting speech understanding and turn predictability, which may be reflected in an altered speech behavior as well as movement behavior during conversation. We also anticipated that these effects would be more pronounced in the presence of high background noise levels, based on the assumption that normal-hearing participants tend to rely more on auditory cues than visual cues in quiet environments, but more on visual cues in noisy environments where verbal interaction is limited and visual communication becomes more important. To quantify the effects, we analyzed measures which were shown in the literature to be affected either by noise or by non-verbal cues. The speech behavior can be divided into voice-related features and timing of speech. One candidate is vocal effort, which was shown to be related to the success of conversations (Beechey et al., 2018). Changes in vocal effort can be measured in terms of the Lombard effect (Brumm & Zollinger, 2011). In face-to-face conversations, an increase in speech level of 3–4 dB per 10 dB increase in noise level is typically observed (Hadley et al., 2019, 2021). Related to the timing of speech, it was found that utterance duration was shorter in high background noise during face-to-face conversation, which may reflect the strategy to share less or simplified information in adverse conditions (Hadley et al., 2019, 2021). In contrast, utterance duration was found to increase in noise in a dyadic puzzle task (Beechey et al., 2018). A facilitated ability to predict the time points of speaker turns is expected to lead to shorter transfers of the speaker floor, referred to as floor transfer offsets (FTOs) (Hadley et al., 2021; Heldner & Edlund, 2010; Levinson & Torreira, 2015).

Related to head movement behavior, with increasing level of head movement animation we expected a reduced search behavior in head movements (Hadley & Culling, 2022). This may be expressed by a smaller angular distance between the participant's head orientation and the currently active avatar. Additionally, the participant's head orientation range was expected to increase with increasing level of animation. The head orientation range represents the range between the two most prominent orientation angles, which were expected due to two conversation partners. Next the head orientation, listeners tend to lean-in closer to the interlocutors in noisy conditions (Hadley et al., 2019). Similarly, a reduced level of head movement animation could also lead to more leaning-in behavior.

With an increased level of head movement animation and an improved turn predictability, an increased perceived conversation success (Nicoras et al., 2023) may be present. Perceived conversation success is a multifaceted concept, subjectively rated by each conversation partner. It reflects both sensory (e.g., hearing ability) and psychosocial (e.g., connection and comfort) dimensions of interaction. Unlike objective methods of measuring conversation success (e.g., based on communication outcome, see Örnolfsson et al., 2023), it can be assessed using a questionnaire.

A virtual environment can be evaluated by the experienced sense of presence by the user. The iGroup Presence Questionnaire (IPQ) (Schubert et al., 2001) comprises the factors of spatial presence, involvement, and the realism of the virtual environment. This questionnaire was also used in a previous study (Hendrikse et al., 2019) to evaluate non-interactive virtual scenes of everyday life, using the same virtual animated characters as in the present study. Here, we expected an increased sense of presence in the virtual environment with an increased level of head movement animation.

## Methods

### Design and Task

**Figure 1.**
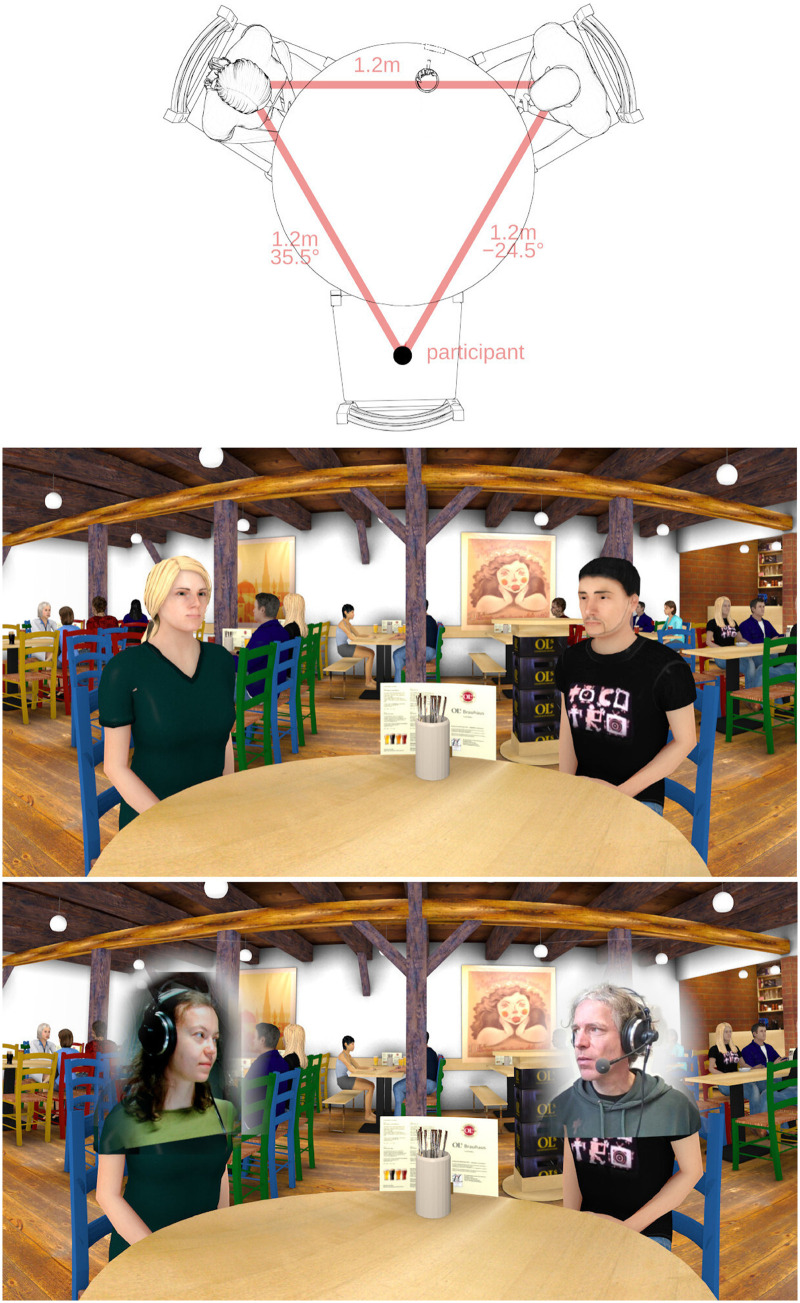
Top panel: top view of the virtual conversation setup. The participant is virtually sitting on the empty chair. Center panel: view of the virtual environment from the participant's perspective. Bottom panel: view of the virtual environment with real-time video textures in front of the avatars’ faces, which was used in the condition “video.”

Free interactive triadic conversations were conducted in virtual reality using telepresence technology. The conversation scenario consisted of three interlocutors, the study participant and two confederates, seated at equal distances around a virtual table in a virtual pub (cf. Figure 1). The confederates were presented with the same virtual acoustic environment, so they experienced noise levels similar to those experienced by the participant in every condition. For the participants, the two confederates were visually represented on a large cylindrical screen by avatars consisting of virtual animated characters with different levels of head movement animation, or by live videos. These live videos were video overlays of the upper torso, inserted into the visual simulation of the scene. They showed the heads and faces on the interlocutors, similar to a video call, while maintaining the virtual surrounding. This setup facilitated the manipulation of the confederates’ head movements. All three interlocutors were seated in different rooms, which allowed access to separate speech and noise audio signals. For the confederates, the participant and the other confederate was visually represented over live video on a computer screen at the correct angular position. The participants as well as the confederates were asked to behave naturally.

Only one participant was invited to each session. The two confederates took part in several sessions. One confederate controlled the ongoing experiment and was referred to as the experimenter. One avatar always represented the experimenter and the other avatar represented a confederate, one of two different people, each taking part in about half of the measurements. The participants were informed before the experiment that the other two interlocutors were the experimenter and a confederate.

To achieve active participation, participants were instructed to maintain a free conversation about casual topics. Picture cards, as suggested by Smeds et al. (2021) and also used by Hartwig et al. (2021), were used to spark the conversation topics, which could freely change, or span multiple conditions. The picture cards were given only to the confederates to keep the focus of participants on the avatars. For a detailed evaluation of avatar head movement effects, multiple levels of head movement realism were implemented together with two contrasting levels of background noise, resulting in a 2 × 4 factorial design, see Table 1. In the visually static condition (“stat”), the avatar's head and gaze were fixed over the center of the table. In the automatic head movement condition (“auto”), the avatar reactively oriented the head and eye gaze toward the defined target angle, cued by speech onset detection (Hendrikse et al., 2018). To include a more complex behavior in one condition, the confederate's head movements were recorded by a motion sensor, transmitted to the visual simulation, and used to control the avatar's head orientation in real-time (“trans”). In the condition which was considered to be closest to a face- to-face conversation, a live video image of the confederate's head and shoulders was transmitted to the position of the avatar in the virtual scene (“video”). In the last two conditions, the movements of the confederates matched the spatial setup in the virtual visual simulation presented to the participants, due to the spatial arrangement of the computer screens and the camera.

**Table 1.** Selected Independent Variables and Levels of Avatar's Head Movement (“Animation”) and Background Noise (“Noise”) in a 2 × 4 Factorial Design.

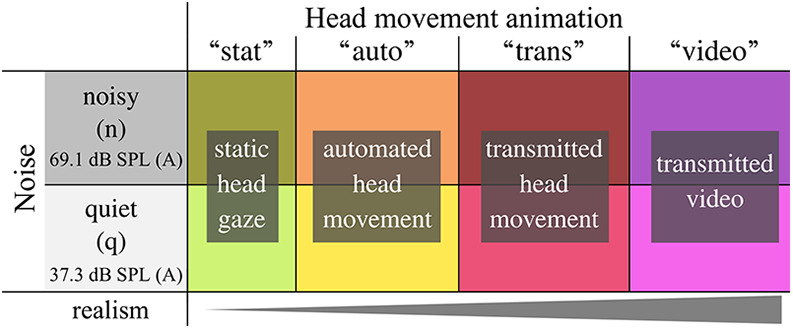

*Note*: The cone indicates the qualitative magnitude of the expected level of realism.

Each audiovisual condition lasted at least 5 min and was manually switched to the next condition by the experimenter at an appropriate time to avoid abrupt interruptions of the conversation. The order of conditions was pseudo-randomized in advance. After each condition, participants completed a questionnaire in which they rated their experience during the last conversation.

### Study Participants

Sixteen participants (nine female, seven male) were acquired via a university-wide online advertisement. The study was approved by the Commission for Research Impact Assessment and Ethics of the Carl von Ossietzky University of Oldenburg (approval number EK/2021/068), and participants were reimbursed for their time. Their age ranged from 19 to 32 years (mean 23.8 years). They stated to have no hearing impairment, and language skills at or close to a native speaker level in German, the language of conversation. Familiarity with virtual reality was not required or assessed. Vision was corrected to normal if necessary. One participant was acquainted with both the experimenter and the confederate before the experiment, one participant with one of them, and 14 participants with neither. The confederates also met the inclusion criteria of the study participants.

### Measurement Setup

**Figure 2.**
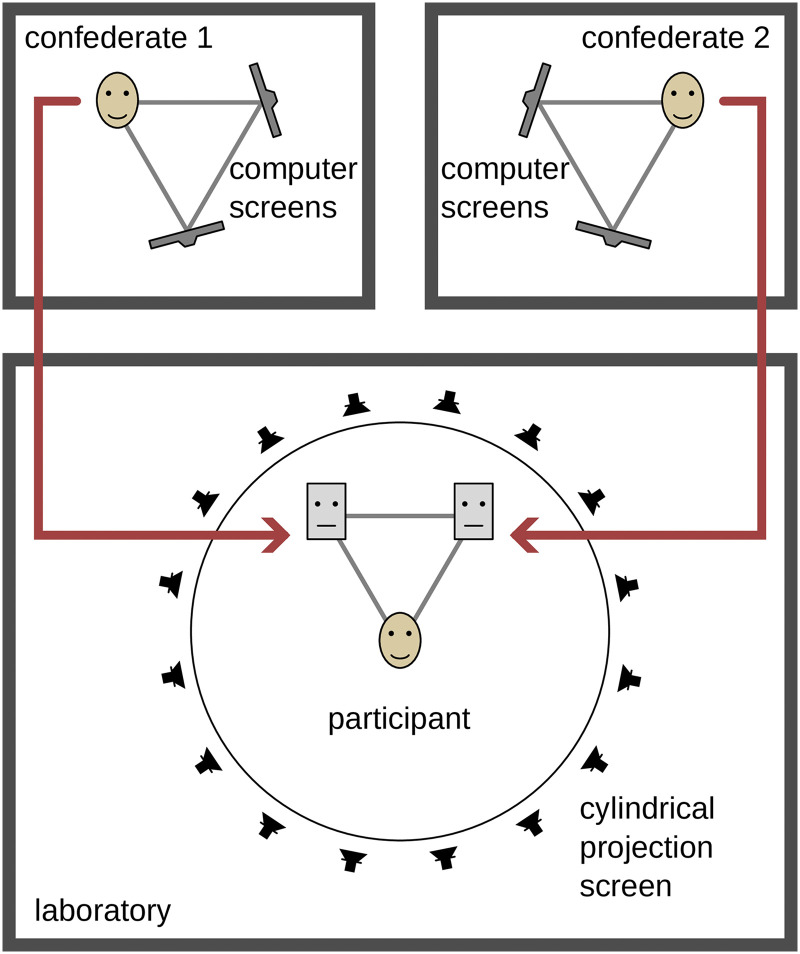
Overview of the setup. The study participant was seated in the laboratory, surrounded by loudspeakers behind a cylindrical screen. The confederates were placed in separate rooms. For the participant, they were represented as avatars. The audio signals were transmitted in low-delay real time. The spatial representation was rendered using a low-delay real-time virtual acoustics engine. Depending on the measurement condition, the head movements or a live video image was transmitted from the confederates to the avatars.

The measurement setup was distributed over three rooms: one for the participants with an audiovisual reproduction using loudspeakers and a cylindrical video projection, and one for each confederate, with binaural headphone reproduction and video reproduction on computer screens (c.f. Figure 2).

*Reproduction setup.* Study participants were seated in a laboratory, surrounded by 45 full-range loudspeakers (Genelec 8020D; 16 at ear level on a circle with a diameter of 3.7 m, 6 at −30*°* elevation, 16 at +15° elevation, 6 at 45° elevation and one at 90° elevation) and a 300° field of view video projection (three NEC U321H, at −105°, 0° and 105°, each with a field of view of 120° and a blending overlap of 15°) on a cylindrical, acoustically transparent projection screen (Heeren et al., 2023). Acoustic and visual rendering were performed on separate computers connected via a network. The audio system was run at a sampling frequency of 48 kHz using block-based processing with a block size of 128 samples for seven participants and 256 samples for nine participants (the block length was increased to avoid artefacts due to dropouts). The Toolbox for Acoustic Scene Creation and Rendering (TASCAR, version 0.228, Grimm, Luberadzka, et al. (2019)) was used to reproduce the virtual acoustics, with vector-base amplitude panning (Pulkki, 1997) used for rendering to loudspeakers. The visual model was reproduced with the Blender game engine (version 2.79b; Roosendaal, 2017), with a virtual camera at the position of the participant. The blending and warping was processed on the graphics card (NVIDIA Quadro M4000). To increase the immersion in the virtual environment, the horizontal position of the receiver and camera in the audiovisual simulation was updated according to the participant's head position, allowing the participant to move in the audiovisual environment. To minimize the number of sensors on the participants, their speech was recorded using a directional room microphone (Sennheiser MKE600) installed at the top of the projection screen. In order to maximize the signal-to-noise ratio of the microphone signal and minimize echoes from the confederates, the transfer functions from each loudspeaker to the room microphone were recorded for static echo cancellation before each session.

The two confederates were seated in separate rooms. Each confederate wore a headset (AKG HSC 271) equipped with a head tracking sensor, see section *Motion capture* for details. The speech signal of the confederates was picked up by a headset microphone and high-pass filtered (*f_cut_* = 120 Hz) to compensate for the near-field effect. The confederates saw the participants and each other via cameras on a computer screen (Dell P2422H, 0.61 m screen diagonal), one for each person. Web cameras (Logitech C270 HD) were mounted on the computer screen and centered between the avatars so as not to cover them. The arrangement of computer screens formed an equilateral triangle between the confederate and the two computer screens to represent the viewing angles and to elicit correct head orientation angles in the confederates. The experiment was controlled via a graphical interface on a laptop computer by one of the confederates, the experimenter.

The laboratory where the participant was seated was acoustically treated with absorbers on the ceiling and walls, a carpet on the floor and a heavy stage backdrop around the spherical loudspeaker array. The resulting reverberation time of the laboratory was below 0.2 s for all frequencies above 500 Hz and below 0.4 s for 125 and 250 Hz. The Direct-to-Reverberant Ratio was increasing from −3.7 dB at 125 Hz to 8.9 dB at 4 kHz, measured from the frontal reproduction loudspeaker at the listening position. One of the confederates’ rooms was another acoustic laboratory and the third was an office.

*Acoustic calibration.* The loudspeakers were calibrated with pink noise in the frequency range 80 Hz–10 kHz. The frequency response of the loudspeakers and the room, measured in one-third octave bands and averaged over three bands, was calibrated by fitting four second-order parametric equalizers. The confederates’ headphones were calibrated by subjective comparison of a headphone-reproduced sound source with a real physical source. The real and simulated sources were placed at a distance of 1.2 m from the listener and played a pink noise in the frequency range 80 Hz–10 kHz. The confederates’ microphones were calibrated by achieving the same sound pressure level at the participants’ position in the laboratory at a distance of 1.2 m using continuous spontaneous speech.

*Motion capture.* The participant's head translation and rotation in three dimensions was tracked with a reflective marker crown, captured by six infrared cameras (Qualisys Miqus M3, Qualisys Track Manager). The experimenter's and confederates’ three-dimensional head rotation was recorded by an inertial measurement unit consisting of a gyroscope, an accelerometer and an Arduino processor, which was mounted on the headset. All movement data were tracked with a sampling rate of *f_s_* = 100 Hz.

*Network transmission and data recording.* The distributed setup was realized by transmitting audio, video and motion data over the local university network. The “OVBOX” system (Grimm, 2021, 2024) was used for audio and motion transmission and experimental control. It provided the transfer of audio data between interlocutors, and transmitted head movement data as well as control commands to the acoustic rendering toolbox. Audio data were then included into the scene, head movement data were forwarded to the visual scene, and commands controlled the virtual scene. As an example, for each confederate, the audio signals of the participant's room microphone and the other confederate plus noise from the virtual scene were transmitted uncompressed via network, rendered locally considering the spatial distribution and orientation of interlocutors, and played back over headphones. This setup also enabled a remote control of the experiment by the experimenter. The “OVBOX” system includes a central relay server, which was also running within the university network. In the “OVBOX” system, data is typically transferred using the Universal Datagram Protocol (UDP). Here, a Transmission Control Protocol (TCP) connection was used between the first confederate (experimenter) and the relay server, at the cost of a greater delay, to ensure secure transmission of all control information without potential packet loss. A peer-to-peer live video transmission service (Seguin, 2022) was used for video transmission.

The audio delay from the confederate microphone to the position of the participants was 49.8 ms, at a block size of 256 samples via the TCP. This delay is larger than typical delays reported by Grimm (2024), which is caused by the relatively large block size and the TCP transmission. The delay between the remote head movement and a movement of the projected image of the avatar was approximately 180 ms. The streamed video pictures had a delay from confederate camera to the lab projection screen of 511.9 ms. The audio delay was not adjusted to the visual delay and stayed consistent over all measurement conditions.

Data from the rating questionnaire was collected via a tablet and sent to the data logger. Short term RMS speech levels were measured in each audio block and sent to the data logger. Data collection was centralized on the audio rendering PC in the laboratory where the participant was seated using the data logging module in TASCAR. This allowed all data sources to be synchronized.

### Virtual Environment

The virtual space represented a pub with other guests at several tables in the background (cf. central panel of Figure 1). The dimensions, acoustic characteristics and audiovisual model are based on an existing room, the “Ols Brauwerkstatt” in Oldenburg, Germany (Grimm et al., 2021). The participants were located at a round table together with two other interlocutors at approximately equal distances (*d_mean_* = 1*.*12 m ± 0*.*03 m) to form an equilateral triangle.

In the acoustic model, the confederates were virtually represented by omnidirectional point sources. Noise sounds were first-order ambisonics field recordings of a babble noise in the local university cafeteria during lunchtime and a refrigerator sound (Grimm, Kothe, et al., 2019). The noise sounds were implemented as diffuse sound fields, i.e., with uniform sound levels within a defined volume. Background music (Dokapi, 2015) was played through two simulated loudspeakers in the room, using a physical model based loudspeaker cabinet simulation of a loudspeaker with a resonance frequency of 80 Hz, see the documentation of the module “spksim” in Grimm (2022) for details. To achieve a higher level of immersion, early reflections and late reverberation of the voices of the confederates and the participants were simulated. The measured broadband reverberation time (T30) at the listening position in the laboratory for a source at the interlocutor's position was 1.27 s (early decay time: 0.17 s), and the Direct-to-Reverberant Ratio was 7.1 dB. In the “quiet” conditions, the refrigerator sound ensured a steady noise floor level at 37.3 dB SPL (A) (48.2 dB SPL (C)) to mask the varying ventilation noise of the projectors. In “noise,” the sounds fridge, babble and music resulted in an *L_eq_* of 69.1 dB SPL (A) (71.1 dB SPL (C)) at the participant's position. All three interlocutors experienced noise according to their position in the virtual environment.

In the visual simulation of the virtual environment, the avatars were blinking at random times and moved slightly back and forth to indicate breathing. Their eye gaze was either directed horizontally over the center of the table (condition “static”) or toward the other avatar's or participant's head (conditions “auto” and “transmitted”). When the avatar's head crossed the central angle between the two interlocutors, its gaze shifted from one to the other. This gaze behavior was intended to simulate typical human behavior without conveying additional information. While the confederates were speaking, the avatar's lips moved in real time via a speech-based algorithm (Llorach et al., 2016). The virtual characters had no facial expression, gestures or body movement. For the “video” condition, a real-time video of the confederate's shoulders and head was streamed to the Blender scene and shown on a flat texture in front of the avatar's face, see lower panel of Figure 1. The virtual characters in the background were only visible during noisy conditions.

### Measures and Analyses

The communication behavior of participants was evaluated regarding speech and head movement. The subjective experience of participants was assessed via a rating questionnaire. For each selected measure, cf. Table 2, one data point per participant and condition was calculated. Variance within conditions was not analyzed, except for the head orientation measure, where range was used as a separate measure.

**Table 2.** Overview of the Dependent Variables and Corresponding Measures.

Variable	Measure	Sensor/Question
Communication behavior:		
Speech behavior	Speech level Utterance duration Speech gap duration Speech overlap duration	mic mic mic mic
Head movement behavior	Angular distance to speaker Head orientation range Head translation	mocap mocap mocap
Experience:		
Sense of presence	Presence in virtual space Copresence with interlocutors Lack of awareness of real env. Realism of virtual world Realism of avatars Realism of voice	Q1 Q2 Q3 Q4 Q5 Q7
Perceived conversation success	Being spoken to in a helpful way Being able to listen easily Sharing information as desired Making an effort to be understood	Q6 Q8 Q9 Q10

*Note*: Behavior was assessed using physical sensors: speech behavior was based on level analysis of head-mounted or room microphones (mic), and movement was measured using an optical motion capture system (Mocap). A questionnaire was used to quantify the sense of presence and the perceived conversation success, see Table 3 for the presented questions (Q1…10) and their translations.

**Table 3.** Questionniare Items That Were Asked After Each Condition.

	Presented Question	English Translation	Verbal Response Anchors
Q1	“Ich hatte das Gefühl, in dem virtuellen Raum anwesend zu sein.”	“I felt present in the virtual space.”	“not true at all” —“completely true”
Q2	“Ich hatte das Gefühl, mit den Gesprächspartnern in dem virtuellen Raum zu sein.”	“I had the feeling of being in the virtual room with the conversation partners.”	“not true at all”—“completely true”
Q3	“Der reale Labor-Raum war mir nicht mehr bewusst.”	“I was not aware of my real environment anymore.”	“not true at all”—“completely true”
Q4	“Die virtuelle Umgebung habe ich wie eine reale Umgebung erlebt.”	“The virtual world seemed to be a real world.”	“not true at all” —“completely true”
Q5	“Wierealerschien Ihnen das Aussehen der Gesprächspartner?”	“How real did the appearance of the conversation partners seem to you?”	“not real at all”—“neither nor”—“completely real”
Q6	“Ich wurde in einer Weise angesprochen, der ich gut folgen konnte.”	“I was being spoken to in a helpful way.”	“not true at all”—“completely true”
Q7	“Wierealerschien Ihnen die Stimme der Gesprächspartner?"	“How real did the voice of the conversation partners seem to you?”	“not real at all”—“neither nor”—“completely real”
Q8	“Es fiel mir leicht, in diesem Gespräch zuzuhören.”	“I was able to listen easily.”	“not true at all”—“completely true”
Q9	“Ich konnte Informationen wie gewünscht teilen.”	“I was able to share information as desired.”	“not true at all”—“completely true”
Q10	“Ich musste mich anstrengen, um besser verstanden zu wer- den.”	“I had to make an effort to be better understood.”	“not true at all”—“completely true”

*Note*: The presented questions were in German, consistent with the language of conversation. The English translation is only used for documentation. Answers were given on a 7-point scale (−3 to +3, verbal anchors at ends and center) with the option to rate in 0.5 steps.

*Speech behavior.* The speech analysis was based on short-term levels, which were calculated as mean-square in blocks of 2.9 ms or 5.8 ms length (after 7 participants, the block length was increased to avoid artefacts due to dropouts). These short-term levels were smoothed with a 10 Hz first-order Butterworth low-pass filter and then converted to dB SPL. As in Hadley et al. (2021), short-time levels were smoothed with a rolling 100 ms Hann window. For a robust speech activity detection, a noise floor estimation was done by fitting a linear model to the level distribution. The level thresholds for speech activity were set to the 25% point of the estimated dynamic range above the noise floor for every person and each condition individually. Speech activity was then defined as all speech levels above threshold. If there was a pause between sections shorter than 1.25 s, the two sections were combined into a single segment, following Hadley et al. (2021). All activity sections shorter than 0.05 s were discarded. The resulting merged segment is referred to as one utterance.

**Figure 3.**
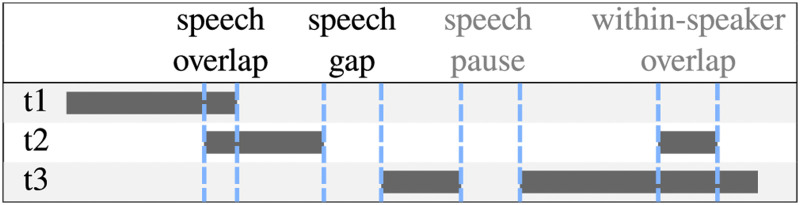
Scheme of possible combinations of speech segments in a triadic conversation. Each row represents one speaker, the dark gray bars are speech activity over time. Gaps and overlaps are marked by light blue bars. Only the times of speech gaps and overlaps were defined as turn takes.

The average *speech level* was calculated by taking the 95th percentile of speech levels during detected speech activity for each participant. For validating the setup in terms of symmetry between interlocutors, also the speech levels of the experimenter and confederate was analyzed. *Utterance duration* was the median duration of all speech activity segments. *Speech gap duration* and *speech overlap duration* were based on the interaction of interlocutors, i.e., based on the time-aligned speech level data, and defined according to Heldner and Edlund (2010), see Figure 3. At speaker turn takes, speech activity pauses or simultaneous activity was detected and the median duration of the respective segments was calculated. The speech overlap duration was noted in negative values, speech gap duration at turn takes in positive values. For a comparison with FTO as reported in the literature, histograms and median values of all FTOs pooled across all participants were calculated for each condition.

*Head movement behavior.* The *angular distance* to the speaker was defined as the median value of absolute angle distances between the participant's head rotation angle and the angle toward the center of the avatar's face around the *z*-axis (i.e., the head yaw angle). The reference points for the avatars’ faces were located between their eyes and mouth, derived from their position in the condition “static.” Similar measures are often used in literature (Hadley et al., 2019; Hendrikse et al., 2022). Only time intervals during which the respective avatar's interlocutor was speaking and the other interlocutors were silent were considered. For an overall measure representing both avatars, the median yaw angle to the corresponding avatar over all conditions was subtracted from the angular distances before calculating the cumulative median. This was done to account for a systematically smaller angular distance to the avatar of the experimenter by most participants.

The *head orientation range* was represented by the difference between the 5th and 95th percentiles of the yaw angle. This was calculated separately for time intervals during which the study participant was listening and speaking. Note that it is independent of which confederate is speaking and is therefore different from angular distance. Furthermore, opposed to the angular distance, this measure captures overshoots. To find individual behavioral changes, the difference of absolute values to the median over all conditions was calculated.

Despite their similar definitions, both measures—angular distance and head orientation range while listening—were retained for the analysis. This allowed for better comparisons with prior literature and captured the differences between speaking and listening time intervals.

The *head translation* of participants was calculated as the median position in the direction toward the center between the two virtual characters. Here again, the difference of absolute values to the median over all conditions was calculated to find individual changes.*Experience rating.* The *rating questionnaire* was a set of 10 questions on a 7-point rating scale with verbal anchors at the floor, center, and ceiling value. Six questions were inspired by the igroup presence questionnaire (IPQ, Schubert et al. (2001)) which evaluates the experienced presence, involvement and realism of a virtual environment. Four items were questions based on the clusters of perceived conversation success identified by Nicoras et al. (2023). The questions were first phrased in English based on the proposed clusters, and then translated into German. The questionnaire items and their respective anchors are listed in Table 3. The questionnaire collected one rating value from each participant for each question and condition. The resulting data points were not pooled or processed further before the statistical analysis.

In addition, a factor analysis was conducted to examine the effects of animation level and background noise on the latent constructs “sense of presence” and “perceived conversation success.” For each construct, a one-factor model was tested using the respective items: Q1–Q5 and Q7 for sense of presence, and Q6, Q8–Q10 for perceived conversation success. Values were pooled across all participants. Factor loadings were estimated using the regression method for factor score prediction, with no rotation applied to maintain interpretability of the single underlying factor.

*Statistical analysis.* A two (noise level) by four (animation level) repeated-measures univariate analysis-of-variance (ANOVA) was performed for the speech and movement behavior data. Sphericity was tested with Mauchly's Test of Sphericity, and in the case of violation the degrees of freedom were corrected with a Greenhouse–Geisser correction factor. A main effect was significant if *p* ≤ *α*, with *α* = 0*.*05. A possible main effect of “animation level” or an interaction effect was further evaluated with a pairwise comparison of the estimated marginal means to obtain the differences between individual levels. The inflation of the family-wise error rate was controlled via the Bonferroni correction as a conservative control over the false rejection of a true null hypothesis. Additionally, the effect size of significant effects was estimated by calculating the partial eta *η*^2^. If a measure was analyzed for all three interlocutors, *interlocutor* was included as a between-subjects factor to find differences between the interlocutors.

For the experience rating data, a Kruskal–Wallis rank sum test was performed, for the factors “animation level” and “noise level.” In case of a significant effect, a Dunn's test with Bonferroni correction of the *p*-value for multiple comparisons was applied for post-hoc analysis to analyze the different levels.

## Results

The study data consists of 16 sessions, with one missing session for head movement data due to a technical error.

### Speech Behavior

The effect of “noise” on participants’ speech level was significant, see Table 4, with a mean increase of 10*.*6 dB in an increase of noise of 22*.*9 dB SPL (C). There was no significant effect of head movement animation level.

**Table 4.** Summary of Main Effects of Communication Behavior as Reported by the ANOVA With *F* and *p* Value, Effect Size *η^2^_p_*.

		**Animation**					**Noise**			
Variable and Measures		*F*	*p*	*η^2^_p_*	pairwise comp.	Δ	*F*	*p*	*η^2^_p_*	Δ
Speech behavior										
	Speech level	3.45	.024	.187	static | video (*p* = .087)		692.27	<.001	.979	10.6 dB
	**Utterance duration**	3.8	.016	.202	autom | video (*p* = .036)	0.79 s	1.52	.237		
	Speech gap duration	1.71	.179				6.35	.024	.297	0.074 s
	Speech overlap duration	1.11	.354				16.28	.001	.520	0.36 s
Head movement behaviour										
	Head translation	0.74	.482				8.31	.012	.372	3 cm
	**Angular distance to speaker**	4.82	.006	.256	static | video (*p* = .039)	2.09°	14.4	.002	.507	−1.6°
	Head orientation range (listening)	2.31	.090				17.19	.001	.551	3.4°
	**Head orientation range (speaking)**	6.32	.001	.345	static | video (*p* = .047)	5.81°	6.56	.025	.353	3.9°
					autom | video (*p* = .005)	7.58°				

*Note:* Significant pairwise comparisons, and mean increase Δ (noise: in noise compared to in quiet. Animation: in higher compared to lower animation level) are reported. Measures in bold indicate a significant effect of animation level.

When *interlocutor* was used as a between-subjects factor, the three interlocutor datasets were not found to be different, except that the confederate had a slightly lower speech level than the participants (*p*_bonf_ = 0*.*047, mean difference: 1*.*8 dB). In low background noise, the median speech level was at 67*.*6 dB SPL (participant), 65*.*1 dB SPL (experimenter) and 65*.*1 dB SPL (confederate). In noisy conditions, median values increased to 78*.*2 dB SPL (participant), 79*.*2 dB SPL (experimenter) and 77*.*2 dB SPL (confederate).

**Figure 4.**
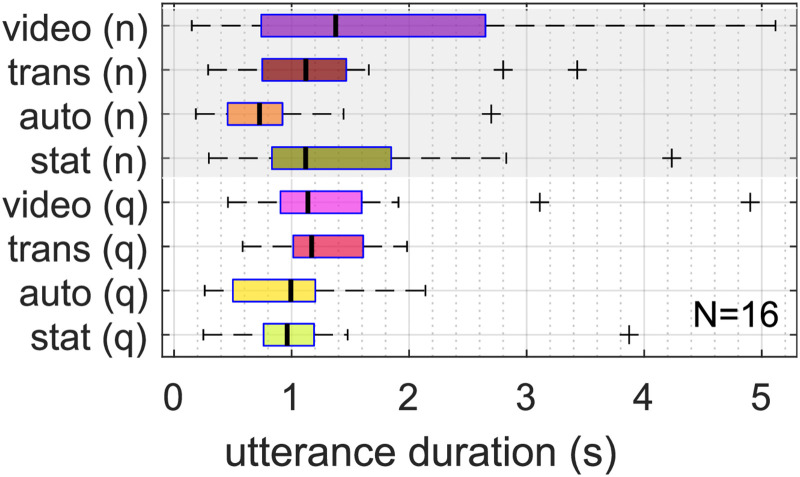
Median utterance duration during conversation.

The median duration of utterances during conversation are shown in Figure 4. Pooled across all conditions, the median duration was 1*.*08 s. The ANOVA revealed that the utterance duration was affected by “animation,” see Table 4, with longer utterances in “video” compared to “automatic” (mean difference: 0*.*79 s). There was no significant effect of “noise” and no interaction effect (*p* = *.*404).

**Figure 5.**
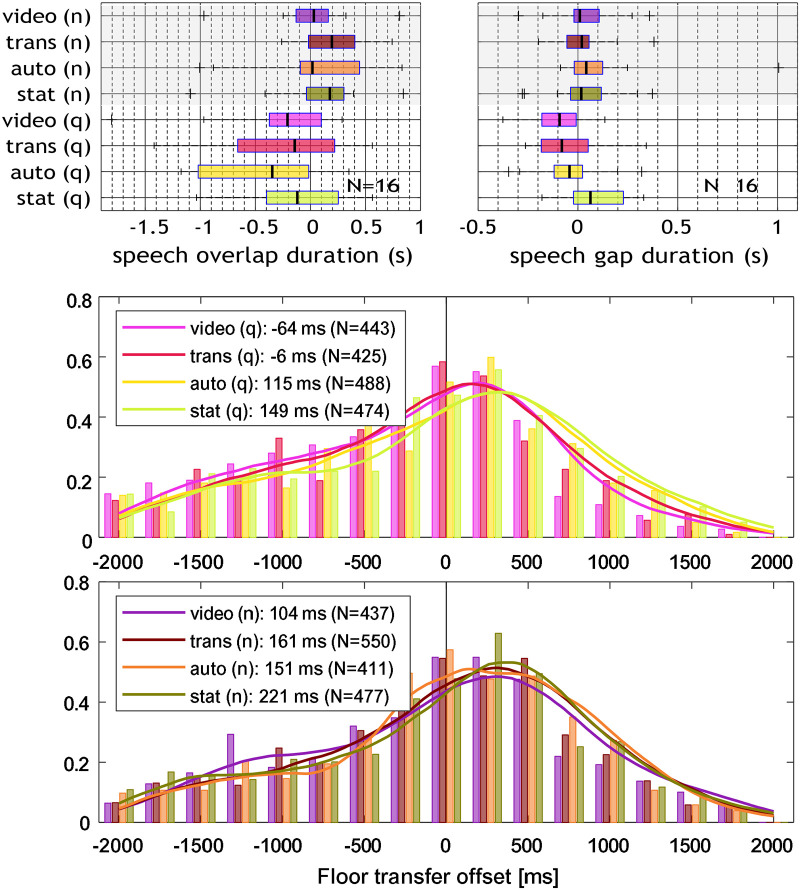
Upper panels: median duration of speech overlaps (left) and gaps (right) at turn takes between the three interlocutors, relative to individual median across all conditions. The speech overlap duration is noted in negative values, i.e., more negative numbers denote a larger overlap. Lower panels: Floor transfer offset (FTO) histogram in 250 ms bins for the quiet (q) and noise (n) conditions, together with a probability density estimate to visualize the shift in median values with animation level and noise. The median FTO and number of floor transfers N is indicated for each condition.

The median duration of speech gaps and overlaps at turn takes are shown in Figure 5, upper panels. For comparison with the literature, histograms of FTOs are presented in the two lower panels. The median duration of all detected speech gaps was 0.51 s, speech overlaps had a median duration of 1.11 s. ANOVA results showed that speech gaps and overlaps were significantly affected by “noise,” but not by “animation,” see Table 4. Speech gap duration increased in noise by 0.074 s in mean value. A possible interaction effect was not significant (*p* = *.*066). Speech overlaps were shorter in noise (mean difference: 0*.*36 s). Additionally, it was found that in noisy conditions, the frequency of speech gaps was increased by Δ*f*_gaps_ = 0*.*72 gaps per minute, i.e., the participants showed fewer overlaps and more gaps at turn takes.

### Head Movement Behavior

**Figure 6.**
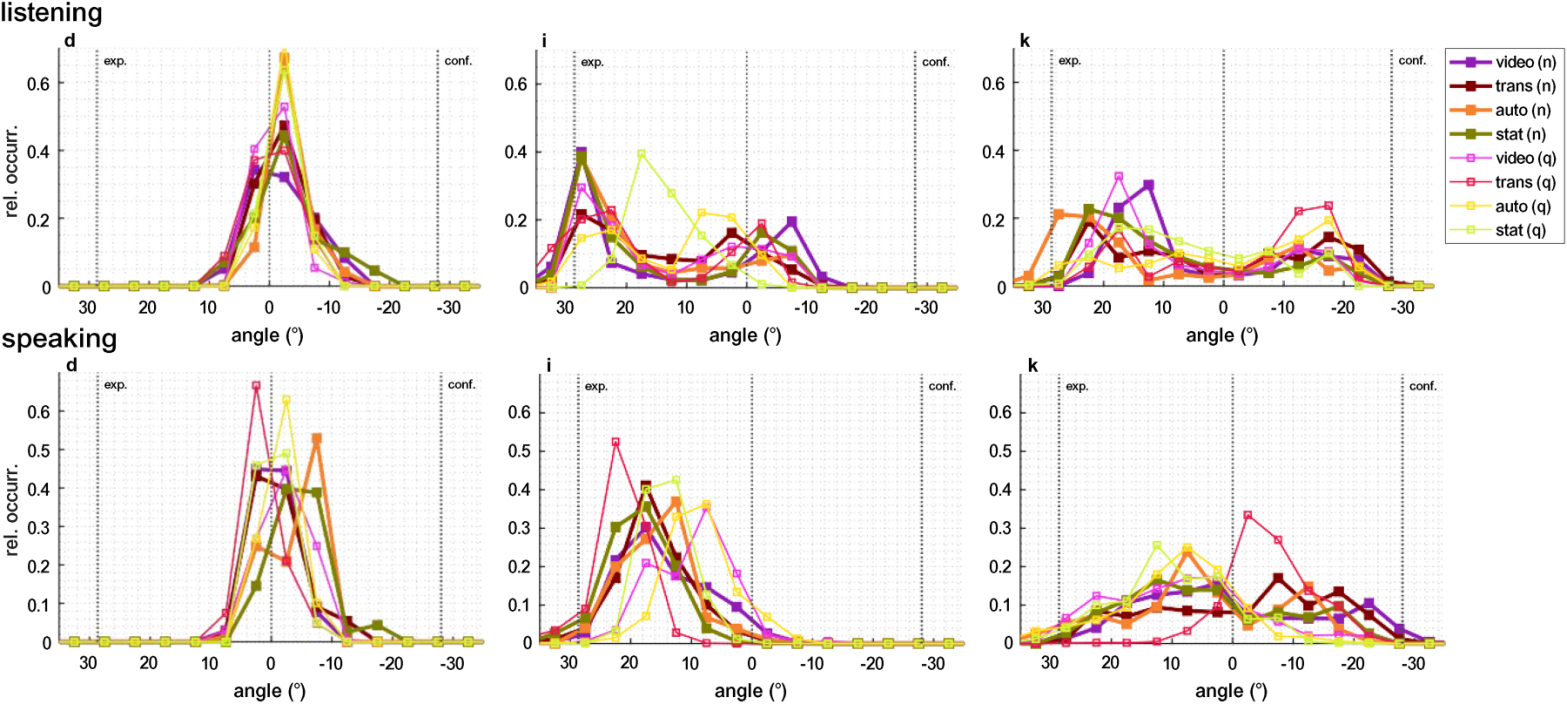
The relative occurrence of the head yaw angle for three typical participants is shown during listening (top panel) and speaking (bottom panel) in all conditions. The vertical lines indicate the positions of the avatars representing the experimenter and the confederate, as well as the center between them. Occurrence values sum to 1 and the angle is divided into 5° increments. During listening, a bimodal distribution can be seen for participants (i) and (k), but not for (d), who did not move their head. Participant (i) oriented the head closer to the experimenter than to the confederate, which may have been caused by a torso orientation that was more toward the experimenter. This may be because, at the beginning of the experiment, the experimenter dominated the conversation by providing general instructions. While speaking, most participants did not exhibit a bimodal distribution.

Participants consistently did not orient their heads completely toward the avatars’ faces, but showed some amount of head angle undershoot. Figure 6 shows histograms of head yaw angle in the different conditions for three participants with typical, yet different, head movement patterns. During the listening phases, some participants exhibited a clear bimodal distribution of yaw angles. Other participants barely moved their heads. In Figure 11 (appendix), individual histograms of head movement angles of every recorded session are displayed. Visually different patterns were found between listening and speaking, mostly pronounced in individuals (f) to (o).

**Figure 7.**
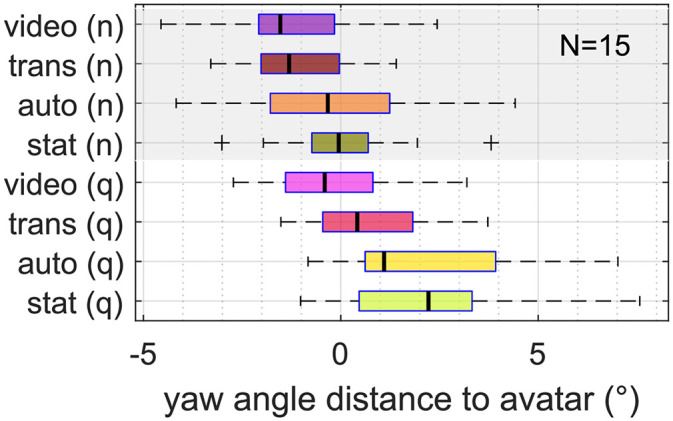
Participants’ angular distance to the actively speaking avatars’ faces in each condition, referenced to the individual median over all conditions. Lower values represent a more accurate orientation; values below zero indicate an orientation closer to the avatars’ faces than the individual baseline. Undershoots and overshoots can not be distinguished, however, most participants did not show overshoots (see Figure 11). One outlier is outside the displayed *x* value range (in video (q): *−*11*.*48°).

The overall median angular distance was not symmetric, participants were more closely oriented toward the avatar range IQR = 1*.*0), where +3*.*0 corresponded to “completely true” and 0 to “neither nor.” The Kruskal–Wallis rank sum test revealed that the rating was not affected by “animation” or “noise,” see Table 5. The participant rating of the awareness of the lab environment (Q3) and the realism of the scene (Q4) was rated with a median value of 1. The Kruskal–Wallis rank sum test revealed that the rating was not affected by “animation” or “noise.” The avatars were rated as significantly more visually realistic (Q5) with higher levels of “animation,” with a significant difference between the video condition and all other conditions. The voice of the conversation partners (Q7) was rated consistently as realistic (median: +3*.*0*,* IQR = 1*.*0). The Kruskal–Wallis rank sum test revealed that the rating was not affected by “animation” representing the experimenter (15*.*4°, median over all conditions and participants) compared to avatar of the confederate (23*.*4°). The pooled data over both avatars, see Figure 7, showed that angular distance changed with levels of head movement animation and in noise. Participants oriented their heads more accurately toward the avatar faces in noise by a mean of 1*.*6° and had a closer orientation in “video” compared to “static” by 2*.*1°, see Table 4.

**Table 5.** Summary of Effects of the Experience Rating as Reported by the Kruskal–Wallis Rank Sum Test, With *χ*^2^ and *p* Value.

		**Animation**				**Noise**		
Variable and Measures		χ^2^	*p*	pairwise comp.	Δ	χ^2^	*p*	Δ
Sense of presence		3.31	.350			1.15	.290	
Q1	Presence in virtual space	2.65	.450			0.97	.320	
Q2	Copresence with conv. partners	3.06	.380			0.98	.320	
Q3	Lack of awareness of real env.	3.5	.320			1.81	.180	
Q4	Realism of virtual world	3.42	.330			3.14	.080	
Q5	**Realism of avatars**	23.7	<.001	static | video	−4.5	0.58	.450	
				autom. | video	−3.8			
				transm. | video	−3.2			
Q7	Realism of voice	4.09	.250			0.17	.680	
Perceived conversation success		3.38	.370			75	<.001	−8.7
Q6	Being spoken to in a helpful way	5.66	.130			13.82	<.001	−3.7
Q8	Being able to listen easily	1.81	.610			76.66	<.001	−8.8
Q9	Sharing information as desired	2.27	.520			27.4	<.001	−5.2
Q10	Making an effort to be understood	2.25	.520			74.33	<.001	8.6

**Figure 8.**
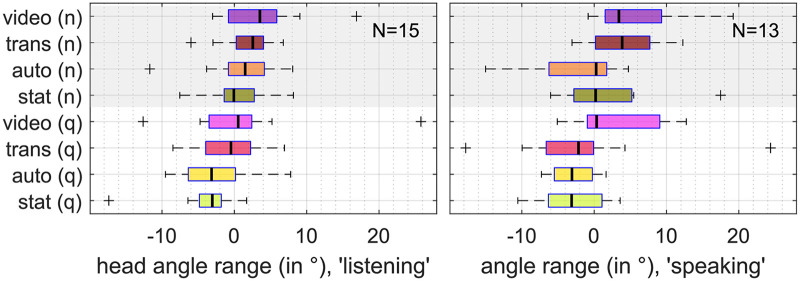
Head orientation range during “listening” (left) and “speaking” (right), referenced to the individual median over all conditions. A higher value represents a wider angle range. Two data sets had a “speaking” period below 15 s in at least one condition and had to be excluded.

The used range of head yaw angles was individually different (cf. Figure 11), individual median values over all conditions during listening or speaking ranged from 11*.*2° to 48*.*3°, with a median of 28*.*2° over all participants. The separate analysis during speaking and listening resulted in different effect patterns, see Figure 8. The ANOVA resulted in a significant main effect of “animation” on the range of head yaw angles during speaking, but not during listening. In noise, head orientation range was increased during speaking and listening, see Table 4.

The head translation toward the screen which displayed the two avatars was affected by noise, see Table 4. Participants positioned their heads 3*.*0 cm closer to the screen in noisy conditions. There was no significant main effect of “animation” and no interaction effect (*p* = *.*366).

### Experience Rating

**Figure 9.**
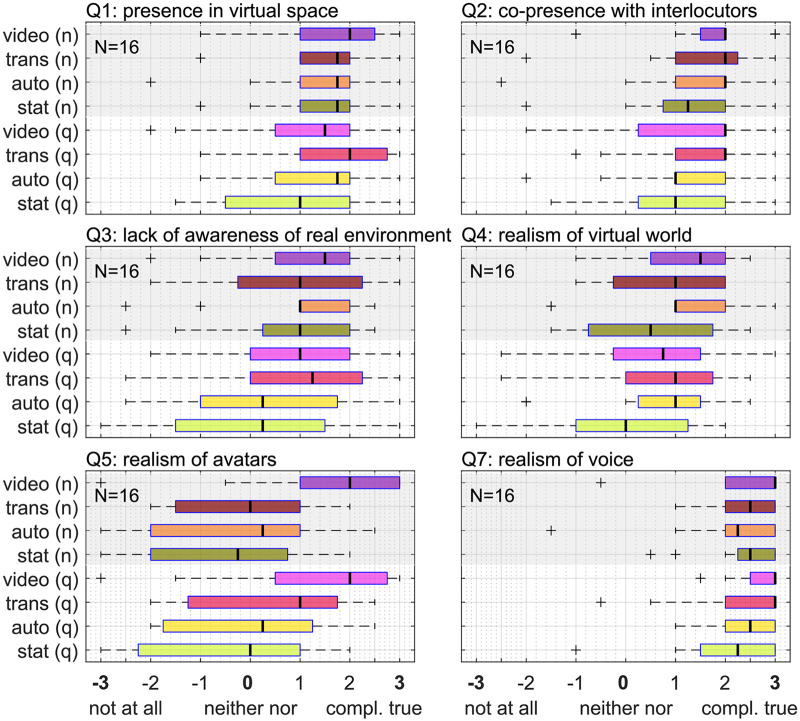
Rating values of questionnaire items regarding presence, involvement and realism of the virtual scene. The English labels are parts of the translated original questions in German (cf. Table 3).

Participants rated their presence in the virtual space (Q1, see Table 3) and their co-presence with their conversation partners (Q2) with a median value of +2*.*0 (interquartile or “noise.” For an overview of the sense of presence related questions see Figure 9.

**Figure 10.**
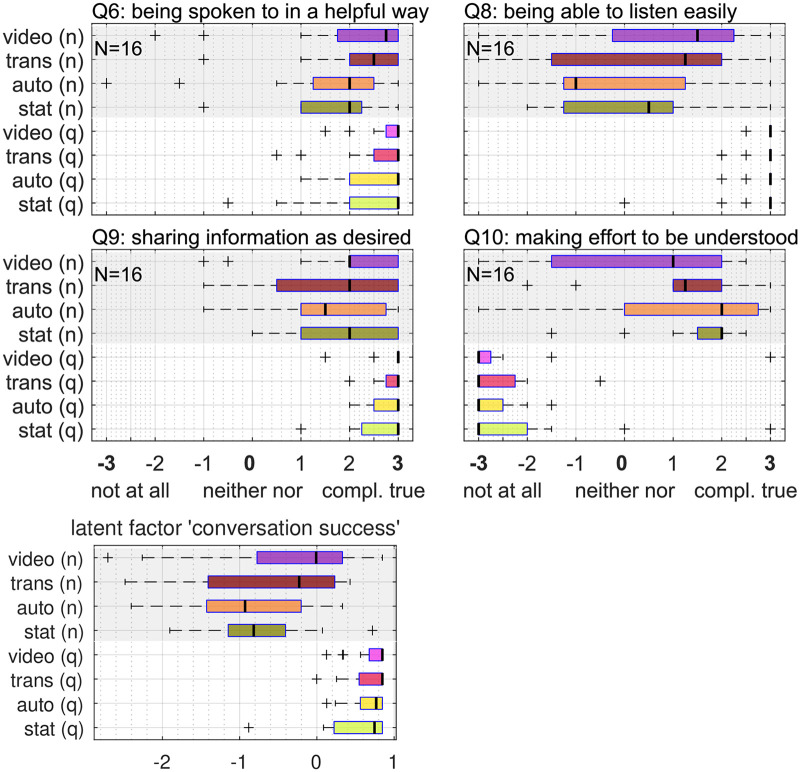
Upper panels: Rating values of questionnaire items regarding perceived conversation success. The English labels are parts of the translation of the original questions in German (cf. Table 3). Note that Q10 has a reversed direction. Bottom panel: latent factor related to “conversation success.”

For all four questions regarding perceived conversation success (see Figure 10), the effect of noise was significant, as shown by the Kruskal–Wallis rank sum test. Participants indicated that they were less spoken to in a helpful way (Q6) and less able to listen easily (Q8). Participants also rated that in noise, they were less able to share information as desired (Q9) and had to make a higher effort to be understood (Q10). Based on the Kruskal–Wallis rank sum test no effect of animation level on the perceived conversation effect could be found.

A factor analysis of the questionnaire data was performed, by splitting the questions into the two constructs “sense of presence” and “perceived conversation success” (see Table 2). The resulting loadings and specific variances of each question are shown in Table 6. Only a question which is not well explained by the common factors shows a significant effect of animation level (Q5; see Table 5). The loading of Q10 is negative, which can be explained by the fact that the scale was reversed for that question (from low effort to high effort), while the other items were ordered from poor perception to good perception. The Kruskal–Wallis rank sum test revealed no significant effect of noise or animation level on the common factor related to “sense of presence.” A significant effect of noise was found for the common factor related to “perceived conversation success” (*F* = 118*.*72, *p* *<* *.*001); the common factor was 1.3 points higher in quiet than in noise.

## Discussion

In this study, we investigated the influence of avatars’ head movements on selected measures of speech behavior and self-motion, and collected feedback via an experience rating questionnaire. We expected an influence of the level of animation on communication behavior in background noise conditions, reflecting strategies to compensate for the degraded acoustic information, and to a lesser extent also in quiet conditions. Furthermore, we expected an improved perceived conversation success with increasing level of animation, because head movement cues provide non-verbal information which can be utilized to predict turn takes (Hadley & Culling, 2022; Templeton et al., 2022).

*Measures of speech behavior.* The participants exhibited an increase in speech level in the presence of higher background noise, which is a reaction known as the “Lombard effect” (Brumm & Zollinger, 2011). The relative increase in speech level of approximately 4.6 dB per 10 dB increase in background noise is comparable to values reported for face-to-face conversations (Hadley et al., 2019, 2021). Speech levels of the participant and the confederate were not different from the experimenter, indicating that the noise level similarly affected difficulty for the participants and the confederates, which is an indicator that conditions were truly symmetric. The mean speech level difference of 1.8 dB between the confederate and the participant may reflect differences in individual speech behavior, a differently mounted headset microphone after calibration, or a calibration mismatch.

The increased level of background noise did not affect utterance duration of the participants, opposed to Hadley et al. (2021), who reported shorter conversational utterances in noise. The observed significant increase in median utterance duration of participants with higher levels of animation can be either caused by overall longer utterances, or fewer short utterances during conversation, such as fewer backchannels, i.e., affirmative utterances (Borch Petersen et al., 2023), during listening. Alongside with the argumentation of Hadley et al. (2021), longer utterances could indicate that, with higher levels of avatar animation, participants shared more complex, i.e., detailed, verbal information. It may be the case that different behavioral patterns were present: for example, long utterances in quiet could contain more complex information, whereas long utterances in noise could be used to keep the conversation going. However this is speculative, as the motivation for longer utterances could not be determined with our data.

Utterance duration values recorded here differed from literature which investigated free face-to-face conversations (median over all conditions here 1.08 s in contrast to Hadley et al. (2021) with values around 4 s). In speech corpora, utterances have a mean duration of about 2 s, independent of language (Gonzalez-Dominguez et al., 2015). Although the participants’ task and analysis approach are considered to be very similar to Hadley et al. (2021), differences in the detection and removal of backchannel utterances may remain, leading to a lower median value of utterance duration. It can be assumed that the speech activity detection algorithm had a considerable influence on this measure.

Although a decrease in median FTO values was present, neither speech gaps nor overlaps between speaker turn takes were significantly affected by head movement animation. It has to be considered that this measure is based on the interaction of all three interlocutors, of which only one person was exposed to the visual conditions. When all three interlocutors are immersed in the visual environment, this effect may be larger. Gaps tended to be shorter with higher animation levels in quiet, but not so much in noise, contrary to expectations, see Figure 5. As improved turn predictability is expected to affect speech gaps (Hadley et al., 2021; Heldner & Edlund, 2010; Levinson & Torreira, 2015), it seems that the non-verbal information did not facilitate the conversation in acoustically adverse conditions. Although head movements have been found to accompany speech prosody and support, for example, syllable identification (Munhall et al., 2004), Saryazdi and Chambers (2022) suggested that in noise, a lack of benefit from real-time visual information may be related to occupied cognitive processing in adverse acoustic conditions when the audio-visual setting is complex, which was the case in this study.

The effect of noise on speech gaps and overlaps was significant: in the noisy conditions, overlaps were shorter and less frequent, and gaps were more pronounced, i.e., longer and more frequent. This indicates that the interlocutors took more time for their responses in higher background noise, which has also been found by Sørensen et al. (2020). Longer speech gaps are associated with increased cognitive load and processing time due to hindered access to verbal information, and possibly indicates a strategy to facilitate communication in noise (Sørensen et al., 2020).

The magnitudes of gap and overlap duration in this study differ from the values reported in the literature. The median duration of speech gaps at turns in conversations found here is 506 ms, which is approximately 150–300 ms longer than the values typically reported (Heldner & Edlund, 2010; Levinson & Torreira, 2015), and the median duration of overlaps is 1108 ms, which is approximately 650–850 ms longer than the median values reported (Hadley et al., 2019; Heldner & Edlund, 2010; Levinson & Torreira, 2015). The literature typically reports on dyadic conversations. However, triads were found to show even shorter turn transition times than dyads, which can be related to an increased competition for the conversation floor (Holler et al., 2021). The differences in the duration of speech gaps and overlaps can be assumed to be due to the level-based detection of speech activity, which is highly dependent on the time constants in level meters—shorter time constants result in more gaps—and the level thresholds—higher thresholds result in more and longer gaps—used to detect the onset of speech. The possible exclusion of gaps and overlaps above a certain duration, e.g., as in dyads in Borch Petersen et al. (2023), was not considered here. As it is difficult to control for all the parameters involved (background noise level, speech microphone, frequency and spatial characteristics of microphones, and the annotation framework), absolute FTO times cannot be compared across studies. Nevertheless, relative differences between conditions can still be meaningful.

An increased transmission delay (measured: Δ*t* = 49*.*8 ms) compared to the sound transmission in a face-to-face conversation (Δ*t* = 1*.*2 m*/*340 m/s *≈* 3*.*5 ms) was present, and a detrimental effect cannot be excluded. As this delay is much shorter than the delays typically experienced in video conferencing, ranging from 120 to 500 ms (Tsioutas & Xylomenos, 2022), it was anticipated that it would have a much smaller detrimental impact compared to such video conferencing settings. However the extent to which the delay of visual cues influenced behavior remains unclear. The delay of 130 ms in the head movement animation compared to the audio signal, as well as the delay of approximately 512 ms the video overlays appeared was not noticeable Since head movements are relatively slow, a delay of 130 ms in the animation compared to the audio signal was not noticeable to the experimenters. The participants did not appear to notice the delay of approximately 512 ms of the video overlays. However, it may have disrupted audiovisual integration and therefore affected the results.

**Figure 11.**
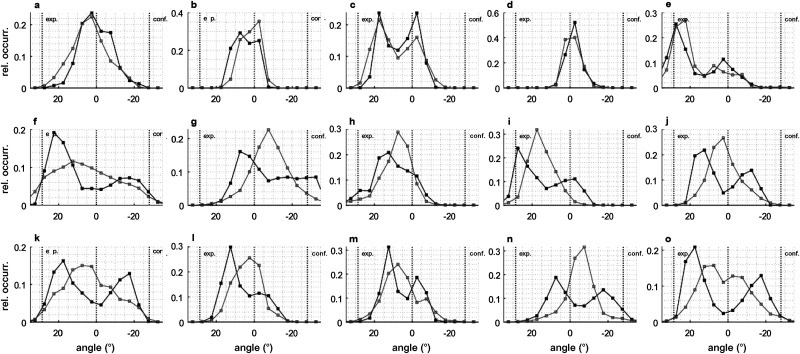
Relative occurrence over head yaw angle for each participant during listening (dark gray, filled symbols) and speaking (light grey, open symbols), pooled over all conditions. The vertical lines indicate the position of the avatar representing the experimenter, the avatar of the confederate, and the center between both. Occurrence values sum up to 1, and the angle is divided into increments of 5°. Definition of listening and speaking: see section Measures and analyses.

*Measures of head movement behavior.* The overall undershoot in head angle to the avatars’ position of 15*.*4° to 23*.*4° is similar to behavior in face-to-face settings (Hadley et al., 2021; Lu et al., 2021). In an immersive virtual audio-visual environment, the behavior of orienting the head only partly toward the target direction seems to be maintained, as also reported by Hendrikse et al. (2019). Most participants oriented their heads more closely toward the experimenter, see Figure 11. The experimenter introduced the participants to the experiment and asked them to complete the questionnaire after each condition. Therefore, increased attention or a shift in posture toward this person seems plausible, although this imbalance was not intended by the study design.

In the video condition, participants oriented their heads 2*.*1° closer to the avatars than in the static condition, possibly because they received more visual information and paid more attention to them. Still, without an analysis of gaze in addition to head movement, this hypothesis cannot be further investigated. The change in head angle distance suggests that how we visually perceive our conversation partners directly affects our head movement behavior. This highlights the importance of using study designs that reflect real-life situations when investigating listeners’ head movement behavior in hearing device research (Grimm et al., 2020). Despite the modest effect found here, we must be cautious in concluding that there is no significant interaction with directional microphones compared to a static visual setup. This is because directional signal processing also affects our orientation patterns (Brimijoin et al., 2014; Grimm et al., 2020), which was not tested in this study.

The effect of background noise level on orientation toward avatars was comparable to the effect of video vs. static, with a mean head angle 1*.*6° closer to the speaker's face in noise. In contrast, Hadley et al. (2021) did not find an effect of speech-shaped noise in a level range of 54 dB–78 dB. It is likely that the slightly more accurate orientation toward the active speaker that was found here was lost in the variance of the interlocutors’ position in Hadley et al. (2021). Our avatars’ heads stayed at the same position, only slightly modulated by the breath animation. This argumentation can also be supported by the results of Lu et al. (2021, Figure 6), who evaluated the head orientation relative to the recorded active talker and found a slightly more accurate orientation of normal-hearing listeners in 70 dB background noise compared to no noise. This indicates that target positions have to be tracked if the goal is to precisely evaluate the listener's orientation.

The qualitative analysis of head orientation ranges (5th–95th percentile of yaw angles) revealed individual differences, ranging from 11*.*2° to 48*.*3° across all conditions. This great individual variance is illustrated in Figure 11 and should be considered in assessing the benefits of directional microphones. Moreover, different head movement behaviors were observed during listening and speaking, consistent with prior studies (Hadley et al., 2019; Hartwig et al., 2021). Most participants had more centered head positions during speaking than during listening (see for Figures 6 and 11), likely to engage with both conversation partners.

The head orientation range increased with the animation level during speaking phases, suggesting a stronger multimodal connection than just acoustic communication. During listening, however, this effect was only evident as a trend. Furthermore, the increase in head orientation range in noisy conditions may indicate a greater reliance on non-verbal cues in adverse acoustic environments.

Participants decreased their distance to the screen in noise by 3.0 cm. This magnitude is consistent with the literature for seated triadic and diadic face-to-face conversations (Hadley et al., 2019, 2021), both resulting in a decrease of inter- personal distance of about 3 cm in an increasing noise level. That indicates that also in the virtual environment participants used this universal strategy, consciously or not, to optimize their acoustic situation, or to indicate difficulties. Due to real-time rendering, which included the subject's head position, leaning-in behavior changed the signal-to-noise ratio (SNR) not only due to proximity to the loudspeakers (which would have a smaller effect than in face-to-face conversations due to the greater distance between physical and virtual sources), but also due to changes in acoustic reproduction position. However, these effects are small in terms of SNR; therefore, we assume that it is more of a social gesture to indicate difficulty.

*Experience of participants.* The rating of avatar realism is plausible, indicating more realistic visual appearance of the conversation partners with higher levels of animation and equally realistic voices. It is worth noting that the participants’ rating of the visual appearance ranged from the negative to the positive end of the rating scale and increased only slightly from absent to transmitted head movement. The detrimental effect of noise on all four questions regarding the perceived conversation success was expected and indicates that the chosen background noise level was adequate to control for perceived conversation success in a normal hearing participant group. The four questions can be grouped into active (Q9, Q10) and passive participation (Q6, Q8).

*General discussion.* We had expected a general effect of avatar head movement animation level on communication behavior and perceived conversation success. We saw this reflected in some of our selected measures: Participants oriented their heads more accurately toward the avatars’ heads, measured by yaw angle distance and overall yaw orientation range during speaking, but did not change the distance between head position and the projection screen. Via the rating questionnaire, participants indicated their conversation partners as more visually realistic. For measures of speech behavior, we cannot confidently conclude a facilitated conversation with higher levels of head movement animation, as speech gaps and overlaps were not affected by animation, and the reason for the increased utterance duration can only be speculated, as discussed above.

For all selected measures, we found most significant differences between the conditions “static” and “video,” rarely between “static” and “transmitted” or “automated” and “video” and never between “automatic” and “transmitted.” This suggests that other facial features, such as eye blinks or displays of emotion, may be more important than realistic head movements. Still, the ANOVA indicated that the effect sizes *η*^2^ of animation on most behavioral measures were moderate (*>*0*.*13) to large (*>*0*.*26), according to Perdices (2018). This indicates that the representation of interlocutors has a considerable influence on the interlocutors’ behavior. Our results also indicate that it may be possible to substitute real head movements with generated movements. Sensible back-channeling of information, e.g., to show attention and to affirm understanding, may be important for a proper involvement of participants into a virtual scene. This behavior can for example be implemented by mimicking the participant's behavior, as suggested by Aburumman et al. (2022).

We had expected a larger effect size of noise compared to animation. This was found for all selected measures of head movement behavior and all but one measures of speech behavior. The effect sizes *η*^2^ of noise on the selected measures, except for utterance duration, were large (*>*0*.*26). Finally, the participants’ conversational behavior in the virtual environment was in part similar to face-to-face conversations. Participants raised their speech level in higher background noise similar to face-to-face conversations (Hadley et al., 2019, 2021), and showed a typical undershoot of head yaw angle to their conversation partners (Hadley et al., 2021; Lu et al., 2021). Without a direct control of face- to-face conversations, the level of behavioral ecological validity in this setup cannot be determined. Still, together with ratings in the upper half of the scale regarding presence, involvement and realism of the virtual scene, the results indicate the potential of high ecological validity in terms of communication behavior, despite the usage of virtual reality and telepresence.

*Limitations.* Speech and head movement behaviors are influenced by the number of interlocutors in a group, hearing status, and age (Hendrikse et al., 2019; Holler et al., 2021; Lu et al., 2021), so the results found here should only be compared to studies investigating triadic conversations of young, normal-hearing participants.

In this study, only the participant was surrounded by the virtual visual environment, which may have reduced the effect of visual conditions on interlocutor interaction, measured here by speech gaps and overlaps at speaker turn takes. Another asymmetry was the confederates’ prior knowledge of the study goals and the experimenter's awareness of the current condition, which may have had an influence on the confederates’ movement, speech levels and between-speaker speech gaps and overlaps.

As noted above, the magnitudes of the selected speech measures were highly dependent on the level threshold for the automatically detected speech activity. Studies using different speech analysis approaches may yield different speech measure values based on the analysis method alone.

The factor analysis revealed that the linear models cannot explain the effect of the animation level, as demonstrated by the large specific variance in the questions that show an individual effect of the animation. This suggests that the questionnaire used in this study was unsuitable to gain insight into the effects of head movement behavior.

One general limitation is the use of virtual reality and animated characters. Participants may have different levels of familiarity with virtual reality. This should be less of a problem when using a projection screen, as in this study. However, it may still affect participants’ willingness to immerse themselves in the virtual environment. Furthermore, animated characters do not display emotions, which could impact the conversations, as indicated by the largest differences being found in the video condition. Conversely, the use of virtual reality and telepresence can be viewed as a positive feature because it enables the explicit modification of salient conversation features.

## Conclusions

In this study, we found that the transmission of head movements to avatars affects the behavior of participants in interactive, triadic conversations in virtual reality. In particular, we found small changes in participants’ head movements, in the duration of utterances, and in the experienced realism of avatars. The effect of transmitted head movements was never significantly different from automated head movements. However, as a general trend, most measures in the transmitted head movement condition fell between those in the video condition and the automated head movement condition. The largest effects were achieved when the video transmission was used to represent the remote interlocutors compared to static avatars.

Therefore, it can be concluded that in the context of interactive communication in virtual reality, the representation of conversation partners will affect the communication behavior of the interlocutor. The participants’ head movement differs between speaking and listening, which is relevant for the evaluation of behavior-based signal processing strategies. Nevertheless, the transmission of head movements alone does not provide sufficient non-verbal communication behavior and further research is needed into the effect of facial expressions, gestures and posture of the interlocutor on communication behavior.

## Appendix

**Table 6.** Loadings of Factor Analysis of the Two Constructs “Sense of Presence” and “Perceived Conversation Success.”

Sense of Presence		
	Loading	Spec. var.
**Q1**	**0**.**95**	**0**.**11**
**Q2**	**0**.**92**	**0**.**16**
Q3	0.50	0.75
Q4	0.65	0.57
Q5	0.44	0.81
Q7	0.37	0.86
Perceived conversation success		
	Loading	Spec. var.
Q6	0.67	0.55
**Q8**	**0**.**90**	**0**.**20**
**Q9**	**0**.**81**	**0**.**35**
**Q10**	**−0**.**76**	**0**.**43**

*Note*: Values in bold indicate questions with a specific variance below 0.5. The common factor of the “sense of presence” questions results in a lowest specific variance for Questions Q1 and Q2 which are directly related to presence, and cannot account for the variance of the questions related realism. the common factor of the questions related to “perceived conversation success” cannot explain the variance of Q6, “being spoken to in a helpful way.”

## References

[bibr1-23312165261432065] Aburumman N. Gillies M. Ward J. A. Hamilton A. F. C. (2022). Nonverbal communication in virtual reality: Nodding as a social signal in virtual interactions. International Journal of Human-Computer Studies, 164(8), 102819. 10.1016/j.ijhcs.2022.102819

[bibr2-23312165261432065] Beechey T. Buchholz J. M. Keidser G. (2018). Measuring communication difficulty through effortful speech production during conversation. Speech Communication, 100(6), 18–29. 10.1016/j.specom.2018.04.007

[bibr3-23312165261432065] Best V. Roverud E. Streeter T. Mason C. R. Kidd G. (2017). The benefit of a visually guided beamformer in a dynamic speech task. Trends in Hearing, 21, 1–11. 10.1177/2331216517722304 PMC5542081 28758567

[bibr4-23312165261432065] Boker S. M. Cohn J. F. Theobald B. J. Matthews I. Brick T. R. Spies J. R. (2009). Effects of damping head movement and facial expression in dyadic conversation using real–time facial expression tracking and synthesized avatars. Philosophical Transactions of the Royal Society B: Biological Sciences, 364(1535), 3485–3495. 10.1098/rstb.2009.0152 PMC2781890 19884143

[bibr5-23312165261432065] Borch Petersen E. Walravens E. Pedersen A. K. (2023). Backchannel behavior in conversations and how it is affected by hearingloss, noise, and hearing aids. In *Forum Acusticum Turin*, pp.2417–2424. 10.61782/fa.2023.0432

[bibr6-23312165261432065] Brimijoin W. O. Whitmer W. M. McShefferty D. Akeroyd M. A. (2014). The effect of hearing aid microphone mode on performance in an auditory orienting task. Ear & Hearing, 35(5), e204–e212. 10.1097/AUD.0000000000000053 PMC4232295 25148290

[bibr7-23312165261432065] Brumm H. Zollinger S. A. (2011). The evolution of the lombard effect: 100 years of psychoacoustic research. Behaviour, 148(11–13), 1173–1198. 10.1163/000579511X605759

[bibr8-23312165261432065] Carr K. Kihm J. (2022). Marketrak—tracking the pulse of the hearing aid market. Seminars in Hearing, 43(04), 277–288. 10.1055/s-0042-1758380 36466564 PMC9715310

[bibr9-23312165261432065] Dokapi. (2015) Lighthouse. URL http://opsound.org/artist/dokapi

[bibr10-23312165261432065] Favre-Félix A. Graversen C. Hietkamp R. K. Dau T. Lunner T. (2018). Improving speech intelligibility by hearing aid eye-gaze steering: Conditions with head fixated in a multitalker environment. Trends in Hearing, 22, 1–11. 10.1177/2331216518814388

[bibr11-23312165261432065] Gonzalez-Dominguez J. Lopez-Moreno I. Moreno P. J. Gonzalez-Rodriguez J. (2015). Frame-by-frame language identification in short utterances using deep neural networks. Neural Networks, 64(4), 49–58. 10.1016/j.neunet.2014.08.006 25242129

[bibr12-23312165261432065] Graf HP Cosatto E Strom V Huang FJ (2002) Visual prosody: Facial movements accompanying speech. In: Proceedings of fifth IEEE international conference on automatic face gesture recognition. IEEE, pp. 396–401. 10.1109/AFGR.2002.1004186

[bibr13-23312165261432065] Grimm G (2021) ORLANDOviols consort box (ovbox). URL: https://ovbox.de

[bibr14-23312165261432065] Grimm G (2022) Hoertech-ggmbh/tascar: Tascar: Release 0.228. 10.5281/ZENODO.7371406

[bibr15-23312165261432065] Grimm G. (2024). Interactive low delay music and speech communication via network connections (OVBOX). Acta Acoustica, 8(18), 1–7. 10.1051/aacus/2024011

[bibr16-23312165261432065] Grimm G Hendrikse M Hohmann V (2021) Pub environment. 10.5281/ZENODO.5886987

[bibr17-23312165261432065] Grimm G. Hendrikse M. M. E. Hohmann V. (2020). Review of self-motion in the context of hearing and hearing device research. Ear & Hearing, 41(Supplement 1), 48S–55S. 10.1097/AUD.0000000000000940 33105259

[bibr18-23312165261432065] Grimm G Kayser H Hendrikse MME Hohmann V (2018) A gaze-based attention model for spatially-aware hearing aids. VDE Verlag GmbH ISBN 9783800747672, pp. 231–235. URL https://ieeexplore.ieee.org/document/8578029

[bibr19-23312165261432065] Grimm G Kayser H Kothe A Hohmann V (2023) Evaluation of behavior-controlled hearing devices in the lab using interactive turn-taking conversations. In: Proceedings of the 10th Convention of the European Acoustics Association, Forum Acusticum 2023. European Acoustics Association. 10.61782/fa.2023.0127

[bibr20-23312165261432065] Grimm G Kothe A Hohmann V (2019) First Order Ambison- ics field recordings for use in virtual acoustic environments in the context of audiology. 10.5281/ZENODO.13341921

[bibr21-23312165261432065] Grimm G. Luberadzka J. Hohmann V. (2019). A toolbox for rendering virtual acoustic environments in the context of audiology. Acta Acustica United with Acustica, 105(3), 566–578. 10.3813/AAA.919337

[bibr22-23312165261432065] Hadley L. V. Brimijoin W. O. Whitmer W. M. (2019). Speech, movement, and gaze behaviours during dyadic conversation in noise. Scientific Reports, 9(1), 10451. 10.1038/s41598-019-46416-0 31320658 PMC6639257

[bibr23-23312165261432065] Hadley L. V. Culling J. F. (2022). Timing of head turns to upcoming talkers in triadic conversation: Evidence for prediction of turn ends and interruptions. Frontiers in Psychology, 13(12), 1–14. 10.3389/fpsyg.2022.1061582 PMC9807761 36605274

[bibr24-23312165261432065] Hadley L. V. Whitmer W. M. Brimijoin W. O. Naylor G. (2021). Conversation in small groups: Speaking and listening strategies depend on the complexities of the environment and group. Psychonomic Bulletin & Review, 28(2), 632–640. 10.3758/s13423-020-01821-9 33051825 PMC8062389

[bibr25-23312165261432065] Hartwig M Hohmann V Grimm G (2021) Speaking with avatars - influence of social interaction on movement behavior in interactive hearing experiments. In: 2021 IEEE Conference on Virtual Reality and 3D User Interfaces Abstracts and Work- shops (VRW). Lisbon, Portugal: IEEE, pp. 94–98. ISBN 978-1-66544-057-8. 10.1109/VRW52623.2021.00025

[bibr26-23312165261432065] Heeren J Grimm G Ewert S Hohmann V (2023) Video screens for hearing research: Transmittance and reflectance of professional and other fabrics. arXiv preprint arXiv:2309.11430, 10.48550/ARXIV.2309.11430

[bibr27-23312165261432065] Heldner M. Edlund J. (2010). Pauses, gaps and overlaps in conversations. Journal of Phonetics, 38(4), 555–568. 10.1016/j.wocn.2010.08.002

[bibr28-23312165261432065] Hendrikse M. M. E. Eichler T. Hohmann V. Grimm G. (2022). Self-motion with hearing impairment and (directional) hearing aids. Trends in Hearing, 26, 1–15. 10.1177/23312165221078707 PMC8966140 35341403

[bibr29-23312165261432065] Hendrikse M. M. E. Llorach G. Grimm G. Hohmann V. (2018). Influence of visual cues on head and eye movements during listening tasks in multi-talker audiovisual environments with animated characters. Speech Communication, 101, 70–84. 10.1016/j.specom.2018.05.008

[bibr30-23312165261432065] Hendrikse M. M. E. Llorach G. Hohmann V. Grimm G. (2019). Movement and gaze behavior in virtual audiovisual listening environments resembling everyday life. Trends in Hearing, 23, 233121651987236. 10.1177/2331216519872362 PMC6732870 32516060

[bibr31-23312165261432065] Hogan A. Phillips R. L. Brumby S. A. Williams W. Mercer- Grant C. (2015). Higher social distress and lower psycho-social wellbeing: Examining the coping capacity and health of people with hearing impairment. Disability and Rehabilitation, 37(22), 2070–2075. 10.3109/09638288.2014.996675 25560217

[bibr32-23312165261432065] Holler J. Alday P. M. Decuyper C. Geiger M. Kendrick K. H. Meyer A. S. (2021). Competition reduces response times in multiparty conversation. Frontiers in Psychology, 12(9), 1–12. 10.3389/fpsyg.2021.693124 PMC8481383 34603124

[bibr33-23312165261432065] Hoppe U. Hast A. Hocke T. (2014). Sprachverstehen mit hörgeräten in abhängigkeit vom tongehör. HNO, 62(6), 443–448. 10.1007/s00106-013-2813-1 24633376

[bibr34-23312165261432065] Keidser G. (2016). Introduction to special issue: Towards ecologically valid protocols for the assessment of hearing and hearing devices. Journal of the American Academy of Audiology, 27(07), 502–503. 10.3766/jaaa.27.7.1 27406657

[bibr35-23312165261432065] Kim G. Y. Cho Y. S. Byun H. M. Seol H. Y. Lim J. Park J. G. Moon I. J. (2022). Factors influencing hearing aid satisfaction in South Korea. Yonsei Medical Journal, 63(6), 570. 10.3349/ymj.2022.63.6.570 35619581 PMC9171670

[bibr35a-23312165261432065] Kothe A. Hohmann V. Grimm G. (2025). *Interactive triadic conversations in virtual reality [data set]*. Zenodo. 10.5281/zenodo.15294859

[bibr36-23312165261432065] Levinson S. C. Torreira F. (2015). Timing in turn-taking and its implications for processing models of language. Frontiers in Psychology, 6(6), 1–17. 10.3389/fpsyg.2015.00731 26124727 PMC4464110

[bibr37-23312165261432065] Llorach G Evans A Blat J Grimm G Hohmann V (2016) Web-Based live speech-driven lip-sync. In: 2016 8th International Conference on Games and Virtual Worlds for Serious Applications (VS-GAMES). Barcelona, Spain: IEEE. ISBN: 978-1-5090-2722-4, pp. 1–4. 10.1109/VS-GAMES.2016.7590381

[bibr38-23312165261432065] Lu H. McKinney M. F. Zhang T. Oxenham A. J. (2021). Investigating age, hearing loss, and background noise effects on speaker-targeted head and eye movements in three-way conversations. The Journal of the Acoustical Society of America, 149(3), 1889–1900. 10.1121/10.0003707 33765809

[bibr39-23312165261432065] Munhall K. Jones J. A. Callan D. E. Kuratate T. Vatikiotis- Bateson E. (2004). Visual prosody and speech intelligibility: Head movement improves auditory speech perception. Psychological Science, 15(2), 133–137. 10.1111/j.0963-7214.2004.01502010.x 14738521

[bibr40-23312165261432065] Naylor G. (2016). Theoretical issues of validity in the mea- surement of aided speech reception threshold in noise for comparing nonlinear hearing aid systems. Journal of the American Academy of Audiology, 27(07), 504–514. 10.3766/jaaa.15093 27406658

[bibr41-23312165261432065] Nicoras R. Gotowiec S. Hadley L. V. Smeds K. Naylor G. (2023). Conversation success in one-to-one and group conversation: A group concept mapping study of adults with normal and impaired hearing. International Journal of Audiology, 62(9), 1–9. 10.1080/14992027.2022.2095538 35875851

[bibr42-23312165261432065] Örnolfsson I May T Ahrens A Dau T (2023) How noise impacts decision-making in triadic conversations. In: Proceedings of the 10th Convention of the European Acoustics Association Forum Acusticum 2023, FA2023. European Acoustics Association, pp. 429–432. 10.61782/fa.2023.0720

[bibr43-23312165261432065] Peperkoorn L. S. Becker D. V. Balliet D. Columbus S. Molho C. Van Lange P. A. M. (2021). The prevalence of dyads in social life. PLOS ONE, 15(12), 1–17. 10.1371/journal.pone.0244188 PMC7769262 33370332

[bibr44-23312165261432065] Perdices M. (2018). Null hypothesis significance testing, p-values, effects sizes and confidence intervals. Brain Impairment, 19(1), 70–80. 10.1017/BrImp.2017.28

[bibr45-23312165261432065] Pulkki V. (1997). Virtual sound source positioning using vector base amplitude panning. J. Audio Eng. Soc, 45(6), 456–466.

[bibr46-23312165261432065] Punch R. Hyde M. (2011). Social participation of children and adolescents with cochlear implants: A qualitative analysis of parent, teacher, and child interviews. Journal of Deaf Studies and Deaf Education, 16(4), 474–493. 10.1093/deafed/enr001 21372111

[bibr47-23312165261432065] Rogers S. L. Broadbent R. Brown J. Fraser A. Speelman C. P. (2022). Realistic motion avatars are the future for social interaction in virtual reality. Frontiers in Virtual Reality, 2(1), 750729. 10.3389/frvir.2021.750729

[bibr48-23312165261432065] Roosendaal T and Blender Foundation. (2017) Blender Software. URL https://www.blender.org/

[bibr49-23312165261432065] Saryazdi R. Chambers C. G. (2022). Gesture and reference to objects in the here-and-now: Listeners’ use of gesture cues in quiet and in noise. Journal of Experimental Psychology: Learning, Memory, and Cognition, 48(4), 583–597. 10.1037/xlm0001035 34180698

[bibr50-23312165261432065] Schubert T. Friedmann F. Regenbrecht H. (2001). The experience of presence: Factor analytic insights. Presence: Teleoperators and Virtual Environments, 10(3), 266–281. 10.1162/105474601300343603

[bibr51-23312165261432065] Seguin S (2022) VDO.ninja. https://docs.vdo.ninja/

[bibr52-23312165261432065] Smeds K. Larsson J. Dahlquist M. Wolters F. Herrlin P. (2021). Live evaluation of auditory preference, a laboratory test for evaluating auditory preference. Journal of the American Academy of Audiology, 32(8), 487–500. 10.1055/s-0041-1735213 34965595

[bibr53-23312165261432065] Sørensen AJM MacDonald EN Lunner T (2020) Timing of turn taking between normal-hearing and hearing-impaired interlocutors. In: International Symposium on Auditory and Audiological Research: Auditory Learning in Biological and Artificial Systems. ISAAR, pp. 37–44.

[bibr54-23312165261432065] Templeton E. M. Chang L. J. Reynolds E. A. Cone LeBeaumont M. D. Wheatley T. (2022). Fast response times signal social connection in conversation. Proceedings of the National Academy of Sciences, 119(4), 1–8. 10.1073/pnas.2116915119 PMC8794835 35042815

[bibr55-23312165261432065] Tsioutas K Xylomenos G (2022) Audio Delay in Web Conference Tools. In: Proceedings of the 7th International Web Audio Conference (WAC). Cannes, France.

[bibr56-23312165261432065] Wolters F. Smeds K. Schmidt E. Christensen E. K. Norup C. (2016). Common sound scenarios: A context-driven catego- rization of everyday sound environments for application in hearing-device research. Journal of the American Academy of Audiology, 27(07), 527–540. 10.3766/jaaa.15105 27406660

